# Arabinogalactan Proteins in the Digestive Glands of *Dionaea muscipula* J.Ellis Traps

**DOI:** 10.3390/cells11030586

**Published:** 2022-02-08

**Authors:** Bartosz J. Płachno, Małgorzata Kapusta, Piotr Stolarczyk, Piotr Świątek

**Affiliations:** 1 Department of Plant Cytology and Embryology, Institute of Botany, Faculty of Biology, Jagiellonian University in Kraków, 9 Gronostajowa St., 30-387 Kraków, Poland; 2 Department of Plant Cytology and Embryology, Faculty of Biology, University of Gdańsk, 59 Wita Stwosza St., 80-308 Gdańsk, Poland; malgorzata.kapusta@ug.edu.pl; 3 Department of Botany, Physiology and Plant Protection, Faculty of Biotechnology and Horticulture, University of Agriculture in Kraków, 29 Listopada 54 Ave., 31-425 Kraków, Poland; piotr.stolarczyk@urk.edu.pl; 4 Institute of Biology, Biotechnology and Environmental Protection, Faculty of Natural Sciences, University of Silesia in Katowice, 9 Bankowa St., 40-007 Katowice, Poland; piotr.swiatek@us.edu.pl

**Keywords:** carnivorous plants, cell wall, Droseraceae, transfer cells, wall labyrinth, wall ingrowths, Venus flytrap

## Abstract

The arabinogalactan proteins (AGP) play important roles in plant growth and developmental processes. However, to the best of our knowledge, there is no information on the spatial distribution of AGP in the plant organs and tissues of carnivorous plants during their carnivorous cycle. The Dionaea muscipula trap forms an “external stomach” and is equipped with an effective digestive-absorbing system. Because its digestive glands are composed of specialized cells, the hypothesis that their cell walls are also very specialized in terms of their composition (AGP) compared to the cell wall of the trap epidermal and parenchyma cells was tested. Another aim of this study was to determine whether there is a spatio-temporal distribution of the AGP in the digestive glands during the secretory cycle of D. muscipula. Antibodies that act against AGPs, including JIM8, JIM13 and JIM14, were used. The localization of the examined compounds was determined using immunohistochemistry techniques and immunogold labeling. In both the un-fed and fed traps, there was an accumulation of AGP in the cell walls of the gland secretory cells. The epitope, which is recognized by JIM14, was a useful marker of the digestive glands. The secretory cells of the D. muscipula digestive glands are transfer cells and an accumulation of specific AGP was at the site where the cell wall labyrinth occurred. Immunogold labeling confirmed an occurrence of AGP in the cell wall ingrowths. There were differences in the AGP occurrence (labeled with JIM8 and JIM13) in the cell walls of the gland secretory cells between the unfed and fed traps.

## 1. Introduction

**Figure 1:**
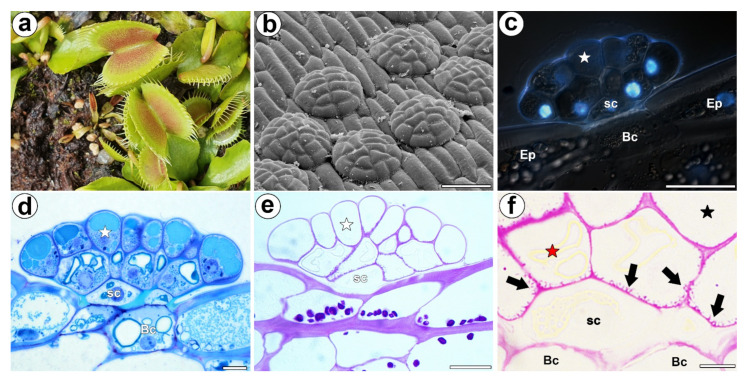
Morphology of a *D. muscipula* trap. (**a**) Traps of *D. muscipula*. (**b**) Morphology of the digestive gland, bar 50 µm. (**c**) Structure of the digestive gland (stained with DAPI, blue fluorescence combined with Nomarski contrast): secretory cell (star), stem cell (sc), basal cell (Bc), epidermal cell (Ep), bar 20 µm. (**d**) A semi-thin section of the digestive gland: secretory cell (star), stem cell (sc), basal cell (Bc), bar 20 µm. (**e**) The digestive gland, PAS reaction: secretory cell (star), stem cell (sc), bar 20 µm. (**f**) The digestive gland, PAS reaction: cell wall ingrowths (black arrows), secretory cell from external layer of gland head (red star), secretory cell from inner layer of gland head (black star), stem cell (sc), basal cell (Bc), bar 2 µm.

*Dionaea muscipula* J.Ellis (Venus flytrap) is a carnivorous plant that creates active traps that are modified leaves and that are used to attract, capture and digest invertebrates, mainly insects, but also spiders [1,2,3]. *Dionaea* was called the “*miraculum naturae*” by Linnaeus [2] and ‘*the most wonderful plant in the world’* by Charles Darwin [4]. Its traps have very sensitive mechanoreceptors and perform very rapid thigmonastic movements [5,6], which is why it has become one of the most common carnivorous plants and a true icon of carnivory in the plant kingdom (it has been used in pop culture, e.g., in the American drama television series “True Blood” and in the anime television series “Maya the Honey Bee”). Since Charles Darwin’s observations, the Venus flytrap *D. muscipula* has been a marvelous research model for studying carnivory in plants. Because of the various processes that occur in the traps (reception of the stimuli needed to close the trap, trap movement, electric signal transmission, enzyme secretion, absorption of compounds from the digested invertebrate bodies), it is a useful model for both physiological and molecular studies [7,8,9,10,11,12,13]. The *Dionaea* trap forms a special external stomach that is equipped with effective digestive-absorbing glands (Figure 1a,b). Each gland consists of basal cells, two stalk (endodermoid) cells and about 32 secretory cells, which form two layers [2] (Figure 1c–f). Each gland cell type is specialized in its ultrastructure and the function that it performs [14,15,16] (and the literature cited therein). It was recently shown that, in *Dionaea*, the mechanical stimulation of trigger hairs creates action potentials that the gland cells translate into touch hormone jasmonate signaling [10,17,18], which triggers exocytosis in the gland cells of the digestive glands [10]. *Dionaea* digestive fluid contains various proteins: peroxidases, nucleases, phosphatases, phospholipases, a glucanase, chitinases and proteolytic enzymes. Some of these are used by ‘typical’ plants as stress pathway-associated proteins. Thus, Schulze et al. [19] proposed that *Dionaea* uses the defense-related processes to form an active digestive system. Moreover, Bemm et al. [9] suggested that the carnivory syndrome in this species evolved via the evolution of an ancient defense pathway, thus replacing cell death with prey digestion and nutrient acquisition. However, for carnivory, other physiological pathways were also used, e.g., Palfalvi et al. [20] suggested that the genes that are used for prey-derived nutrient absorption in *Dionaea* are recruited from the roots.

The *Dionaea* digestive glands not only secrete enzymes and absorb nutrients but they also have the ability to repeat these functions several times [16]. Several authors have postulated that there are changes in the ultrastructure of the secretory cells of the *Dionaea* digestive glands during the digestive cycle [15,21,22,23] and the occurrence of these changes was recently proven in a very elegant way using 3D electron tomography [12,13]. During gland activity, the volume of the cell wall labyrinth space (cell wall labyrinth—the place where cell wall forms wall ingrowths which amplifying plasma membrane surface areas) in the secretory cells changes [15]. Moreover, Boulogne et al. [13] observed an enlarged periplasmic space (space between plasma membrane and cell wall) in these cells during the secretory event (three days after trap activation). These authors suggested that this temporary enlargement of the periplasmic space creates a reservoir for the freshly synthesized enzymes. Thus, the cell walls of the secretory cells are active elements during the digestive-absorptive cycle.

The plant cell wall is composed of a macromolecular network of polymers, mainly cellulose, hemicellulose and pectin but also approximately 10% of the structural proteins, including extensins, glycine-rich proteins, proline-rich proteins, solanaceous lectins and arabinogalactan proteins (AGP) [24]. AGP are hydroxyproline-rich glycoproteins that play important roles in plant cells, including plant growth, the developmental processes and the response to biotic and abiotic stress [25,26,27].

To date, there have been no reports that present the cell wall components of not only the *Dionaea* gland cells but also of the traps of other carnivorous plants. Because the *Dionaea* digestive glands are composed of specialized cells, we tested the hypothesis that their cell walls are also very specialized in terms of their composition in the case arabinogalactan proteins compared to the cell wall of the trap epidermal and parenchyma cells. Another aim of this study was to determine whether there is a spatio-temporal distribution of the AGP in the digestive glands during the secretory cycle of *D. muscipula*. 

## 2. Materials and Methods

### 2.1. Plant Material

The *D. muscipula* plants were purchased from a commercial supplier (GTP Harvey, Baniocha, Poland) and then cultivated in the Department of Plant Cytology and Embryology, Jagiellonian University in Kraków. Plants had been grown without fertilizer in a mixture of sand and peat. The traps were stimulated with blocks of egg white (from a boiled chicken egg) in order to mimic the presence of prey after which the traps were removed. The experiment were done in triplicate. Small portions of the traps with the digestive glands were fixed. We examined unstimulated traps and traps after stimulation (two hours and three days—the secretion phase, five and seven days—the absorption phase).

### 2.2. Histological and Immunochemical Analysis

The detailed procedure for observing the histological sections and conducting the immunochemical analysis were as described in Płachno et al. [28,29]. The plant material was fixed overnight at 4° C in 8% (w/v) paraformaldehyde (PFA, Sigma-Aldrich, Sigma-Aldrich Sp. z o.o. Poznan, Poland) with 0.25% (v/v) glutaraldehyde (GA, Sigma-Aldrich, Sigma-Aldrich Sp. z o.o. Poznan, Poland) in a PIPES buffer. PIPES buffer contains 50 mM PIPES (piperazine-N,N′-bis [2-ethanesulfonic acid], Sigma Aldrich, Poland), 10 mM EGTA (ethylene glycol-bis[β-aminoethyl ether]N,N,N′,N′-tetraacetic acid, Sigma Aldrich, Poland), and 1 mM MgCl2 (Sigma Aldrich, Sigma-Aldrich Sp. z o.o. Poznan Poland), pH 6.8. It was then embedded in Steedman’s wax (for preliminary analysis, using all antibodies). Immunolabelings were repeated twice. For analysis concerning localization JIM14, JIM8, and JIM13 epitopes plant material was embedded in LR White Resin (Polysciences Europe GmbH, Hirschberg an der Bergstrasse, Germany), repeated twice, and sectioned. The rehydrated sections were blocked with 1% bovine serum albumin (BSA, Sigma-Aldrich, Sigma-Aldrich Sp. z o.o. Poznan, Poland) in a PBS buffer and incubated with the primary antibodies anti-AGP: JIM4, JIM8, JIM13; JIM14, JIM15 and MAC207 [30,31,32,33] overnight at 4 °C. All of the primary antibodies were used in a 1:20 dilution and were purchased from Plant Probes, UK and the secondary antibody goat anti-rat conjugated with FITC was purchased from Abcam (Abcam plc, Cambridge, UK). The chromatin in the nuclei was stained with 7 µg/mL DAPI (Sigma Aldrich, Poland) diluted in PBS buffer and the samples were then cover-slipped using a Mowiol mounting medium: mixture of Mowiol ^®^4-88 (Sigma Aldrich, Poland) and glycerol for fluorescence microscopy (Merck, Poland) with addition of 2.5% DABCO (The Carl Roth GmbH + Co. KG, Germany). They were viewed using a Nikon Eclipse E800 microscope equipped with a B-2A filter, a GFP custom filter and a UV-2A, DAPI filter combined with Nomarski contrast (DIC) and Leica DM6000B microscope equipped with a GFP filter. Photos were acquired as Z stacks and deconvolved using 5 iterations of a 3D nonblind algorithm (AutoQuant ™, Media Cybernetics Inc., Rockville, Maryland USA) in order to maximize the spatial resolution, images are presented as the maximum projections. The stacks were obtained using a Leica DM6000B microscope equipped with a GFP filter. At least two different replications were performed for each developmental stage of the analyzed traps and about five to ten sections were analyzed from each organ for each antibody that was used. Negative controls were created by omitting the primary antibody step, which caused no fluorescence signal in any of the control frames for any of the stained slides (Figure S1).

Mean values of fluorescence intensity were calculated from the GFP channel using LAS AF Quantify module (Leica Microsystems). The regions of interest were selected manually to cover 3 secretory cells from 3 different glands in one trap (n = 3). The data were statistically analyzed using Statistica 13 (StatSoft, Poland). For comparisons of the mean values, an analysis of variance (one-way ANOVA) followed by post hoc Tukey’s honestly significant difference test was used. For all analyses, the significance level was estimated at *p*  <  0.05 (supplementary Table S1,S2). Boxplots were created with R ver. 7.0.

Semi-thin sections (0.9–1.0 μm thick) were prepared for LM and stained for general histology using aqueous methylene blue/azure II (MB/AII) for 1–2 min. A histochemical procedure with fixed material using the PAS reaction (the periodic acid-Schiff reaction) was performed to detect the polysaccharides (wall ingrowths) [34].

### 2.3. Immunogold Labeling Distribution of AGP

A Leica Ultracut UCT ultramicrotome was used to prepare the ultrathin sections (50 nm). Ultrathin sections were blocked in 1% BSA (Aurion, Wageningen, The Netherlands) in PBS buffer for 15 min and then incubated in primary antibodies in a 1:10 dilution overnight at 4° C. Next day ultrathin sections were incubated with secondary antibody goat anti rat conjugated with colloidal gold 10 nm (Sigma Aldrich, Poland) in a 1:50 dilution for 2 h, followed by washing in PBS buffer and distilled water. Negative controls were created by omitting the primary antibody step (Supplementary Material 1). Lead citrate (Microshop, Poland) and URANYLess (Microshop, Poland) were added as the contrasting agents. The cells were visualized using a Tecnai Spirit BioTWIN microscope (FEI) at 120 kV (Laboratory of Electron Microscopy, Faculty of Biology, University of Gdańsk).

### 2.4. Morphological Observation

For the SEM, the material was fixed and later processed as described in Lustofin et al. [35], and then dehydrated and dried using supercritical CO_2_. The material was then sputter-coated with gold and examined at an accelerating voltage of 20 kV using a Hitachi S-4700 scanning electron microscope, which is housed at the Institute of Geological Sciences, Jagiellonian University in Kraków, Poland.

## 3. Results

### 3.1. AGP Distribution

**Figure 2:**
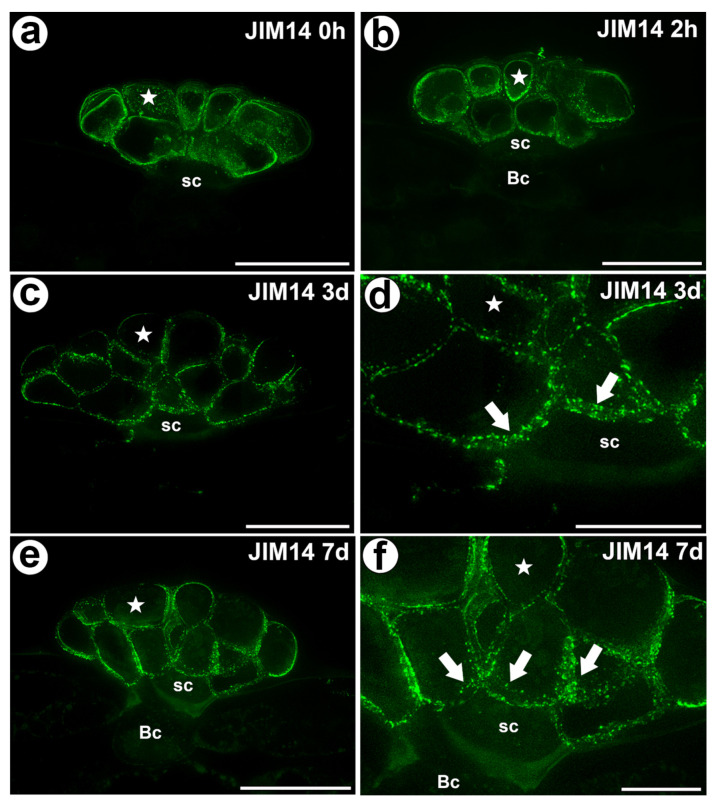
The arabinogalactan proteins (labeled with JIM14) that were detected in a *D. muscipula* trap. (**a**) Immunolabeling of AGP with JIM14 that were detected in the gland from an unfed trap: secretory cell (star), stem cell (sc), bar 20 µm. (**b**) Immunolabeling of AGP with JIM14 that were detected in a gland two hours after feeding: secretory cell (star), stem cell (sc), basal cell (Bc), bar 20 µm. (**c**) Immunolabeling of AGP with JIM14 that were detected in a gland three days after feeding: secretory cell (star), stem cell (sc), bar 20 µm. (**d**) The magnification from (c)—cell wall ingrowths (arrows) in secretory cells (star), stem cells (sc), bar 10 µm (**e**) Immunolabeling of AGP with JIM14 that were detected in a gland seven days after feeding: secretory cell (star), stem cell (sc), basal cell (Bc), bar 20 µm. (**f**) The magnification from (**e**)—cell wall ingrowths (arrows) in secretory cells (star), stem cells (sc), bar 10 µm. All photos are maximum projections from z-stacks.

The epitope that is recognized by the JIM14 antibody was mainly detected in the cell walls of the secretory cells of the digestive glands in both the unfed and fed traps (Figure 2a–f). Because this epitope was abundantly present in these cells, it can be used as a marker of the glands. However, this epitope was absent in the walls of the stalk (endodermoid) cells and the basal cells or only occurred in the basal cell wall where the signal was less intense than in secretory cells.

**Figure 3:**
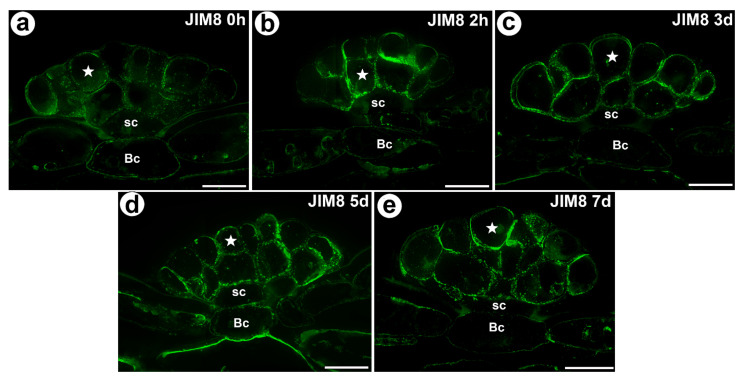
The arabinogalactan proteins (labeled with JIM8) that were detected in a *D. muscipula* trap. (**a**) Immunolabeling of AGP with JIM8 that were detected in the gland from an unfed trap: secretory cell (star), stem cell (sc), bar 20 µm. (**b**) The arabinogalactan proteins (labeled with JIM8) that were detected in a gland two hours after feeding: secretory cell (star), stem cell (sc), basal cell (Bc), bar 20 µm. (**c**) Immunolabeling of AGP with JIM8 that were detected in a gland three days after feeding: secretory cell (star), stem cell (sc), basal cell (Bc), bar 20 µm. (**d**) Immunolabeling of AGP with JIM8 that were detected in a gland five days after feeding: secretory cell (star), stem cell (sc), basal cell (Bc), bar 20 µm. (**e**) Immunolabeling of AGP with JIM8 that were detected in a gland seven days after feeding: secretory cell (star), stem cell (sc), basal cell (Bc), bar 20 µm. All photos are maximum projections from z-stacks.

The AGP epitope that is recognized by the JIM8 antibody was present in all of the cell types of the digestive glands. This AGP epitope was especially abundant in the cell walls of the secretory cells (Figure 3a–e). This AGP epitope also occurred in the cells of the trap parenchyma and epidermis.

**Figure 4:**
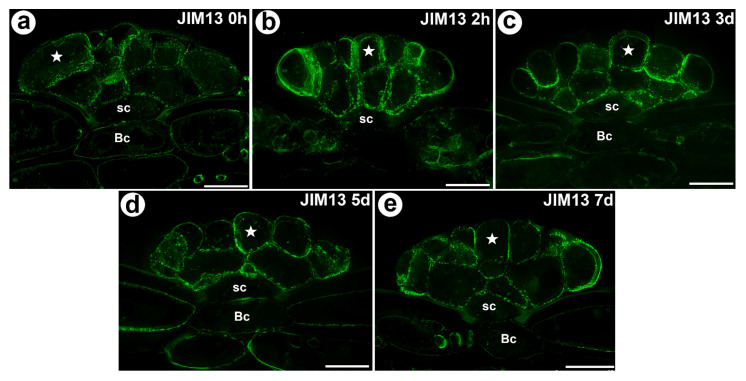
The arabinogalactan proteins (labeled with JIM13) that were detected in a *D. muscipula* trap. (**a**) Immunolabeling of AGP with JIM13 that were detected in the gland from an unfed trap: secretory cell (star), stem cell (sc), bar 20 µm. (**b**) Immunolabeling of AGP with JIM13 that were detected in a gland two hours after feeding: secretory cell (star), stem cell (sc), bar 20 µm. (**c**) The arabinogalactan proteins (labeled with JIM13) that were detected in a gland three days after feeding: secretory cell (star), stem cell (sc), basal cell (Bc), bar 20 µm. (**d**) Immunolabeling of AGP with JIM13 that were detected in a gland five days after feeding: secretory cell (star), stem cell (sc), basal cell (Bc), bar 20 µm. (**e**) Immunolabeling of AGP with JIM13 that were detected in a gland seven days after feeding: secretory cell (star), stem cell (sc), basal cell (Bc), bar 20 µm. All photos maximum are projections from z-stacks.

The AGP epitope that is recognized by the JIM13 antibody had a similar distribution as the AGP epitope that is recognized by the JIM8 antibody (Figure 4a–e). Both of these AGPs occurred in the unfed as well as in the fed traps. None of the epitopes that are recognized by the JIM4, JIM15 and MAC207 antibodies were detected.

### 3.2. Wall Ingrowths Morphology and Immunogold Labeling Distribution of AGP in the Secretory Cells of Digestive Glands

In *Dionaea* secretory cells there were reticulate wall ingrowths (Figure 1f). Wall ingrowths were especially well developed in the secretory cells from inner layer.

**Figure 5:**
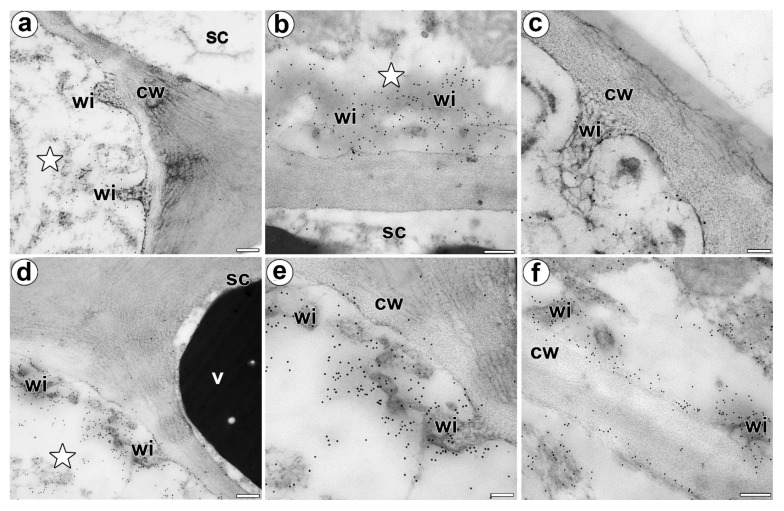
Immunolocalization of the AGP in the cell walls of a *D. muscipula* digestive gland. (**a**) Immunogold labeling of the wall ingrowths with JIM14, a gland from an unfed trap: secretory cell (star), stem cell (sc), wall ingrowths (wi), cell wall (cw), bar 200 nm. (**b**) Immunogold labeling of the wall ingrowths with JIM14, a gland two hours after feeding: secretory cell (star), stem cell (sc), wall ingrowths (wi), bar 200 nm. (**c**) Immunogold labeling of the wall ingrowths with JIM8 in the secretory cell, a gland three days after feeding: wall ingrowths (wi), cell wall (cw), bar 100 nm. (**d**) Immunogold labeling of wall ingrowths with JIM13, in a gland three days after feeding: secretory cell (star), stem cell (sc), wall ingrowths (wi), cell wall (cw), vacuole (v), bar 200 nm. (**e**,**f**) Immunogold labeling of the wall ingrowths with JIM13 in the secretory cells, a gland three days after feeding: wall ingrowths (wi), cell wall (cw), bar 100 nm and bar 200 nm.

In both unfed and fed trap, labeling with anti-AGP (JIM14, JIM8) antibodies was associated with the wall ingrowths (in fibral part and also translucent zone) and periplasmic space in the secretory cells of the digestive glands (Figure 5a–c). Labeling with the anti-AGP (JIM13) antibodies was not only associated with the wall ingrowths and periplasmic space but also with the cytoplasm and the phenolic, osmophilic material in the vacuoles (Figure 5d–f).

**Figure 6:**
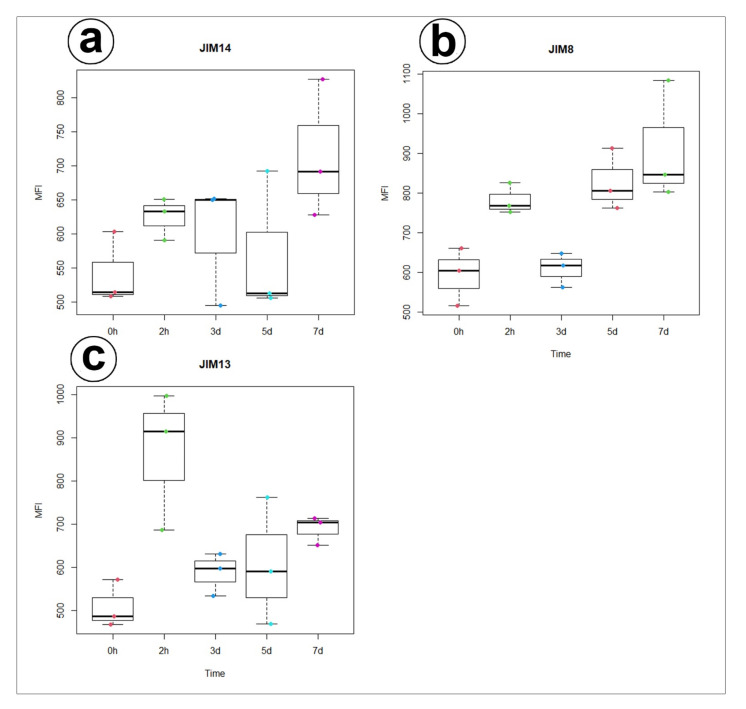
Quantification of immunofluorescence labelling. Mean value of AGP fluorescence intensity (MFI) labelled by (**a**) JIM14, (**b**) JIM8, and (**c**) JIM13 antibodies in 3 secretory cells from 3 different glands (n = 3) during 0 and 2 h, 3, 5, and 7 days after feeding (Time).

Statistical analysis showed no significant differences between AGP labelled by JIM14 in secretory cells of the unfed and stimulated traps. For epitope detected with JIM8 antibody significant differences for mean value of fluorescence signal were noted between unfed (0 h) and traps after 7 days of feeding and significant increase of mean intensity of fluorescence was also observed for traps at 3 and 7 days after feeding. In contrast to epitope detected with JIM13 antibody significant increase of fluorescence were noted between unfed (0 h) and traps after 2h of feeding (Figure 6, supplementary Table S1,S2).

## 4. Discussion

We found a strong accumulation of specific AGP (recognized by the JIM14, JIM8 and JIM13 antibodies) in the digestive gland mostly in the secretory cells. The AGP are known from the vegetative organs (in the mesophyll cell, epidermal cells, xylem, root cap cells, etc.) [36,37], but these specific AGP have commonly been recorded in the reproductive organs where it is believed that they play a role in the communication between generations and are markers for the gametophyte cell differentiation [29,38,39,40]. What could the function of AGPs in the *Dionaea* digestive glands be?

### 4.1. The Secretory Cells of the Dionaea as Transfer Cells

The secretory cells of the *Dionaea* digestive glands are transfer cells [14,16,22]. These types of cells are specialized in high nutrient rates into plant body and are characterized by an intricate wall labyrinth, which supports an increased surface area of the plasma membrane, which is rich in nutrient transporters [41].

Jauh and Lord [42], Vaughn et al. [43], Boughanmi et al. [37] and Henry et al. [44,45] showed the occurrence of AGP in or near the cell wall ingrowths. In the *Dionaea* digestive glands, there was a strong accumulation of specific AGP (recognized by the JIM14, JIM13 and JIM8 antibodies) at the site where the wall labyrinth occurred. We confirmed that AGP occurred in the wall ingrowths (in fibral part and also translucent zone) at level of electron microscopy.

Vaughn et al. [43] proposed that AGP play a role in directing the wall ingrowth deposition. These proteins may provide positional information [46], thus may play role in the polarized formation of the wall ingrowths [41].

There are two main types of wall ingrowth morphology: reticulate and flange [41]. In *Dionaea*, the reticulate type was identified. Wall ingrowths were especially well developed in the secretory cells from inner layer. We think that this is associated with uptake of material (from digestion) and transport of this back to the rest of the plant.

### 4.2. Possible Role of AGP

However, the upregulation of AGP is also connected with the stress that is caused by a high internal hydrostatic pressure [47] or an infection [48,49]. Some authors have proposed that the AGP that are recognized by JIM13 and JIM14 are connected with PCD (programmed cell death) and mark the cells that are destined for PCD [50]. Schulze et al. [19] and Bemm et al. [9] proposed that *Dionaea* uses the stress pathway-related processes to form an active digestive system, which could explain the accumulation of AGP in the digestive glands.

Lamport and Várnai [26] proposed that AGP might act as Ca^2+^ capacitors. Later, using *Arabidopsis* mutants, Lopez-Hernandez et al. [51] proved the significance of the AGP-Ca^2+^ interaction for Ca^2+^ signaling and suggested that the function of AGP is to bind and release the cell-surface apoplastic calcium.

Scherzer et al. [10] showed that the calcium activation of the secretory cells of the digestive glands is therefore crucial for the *Dionaea* trap function. Recently, using transgenic *Dionaea,* Suda et al. [52] linked signal memory to the calcium dynamics in the traps. Thus, an accumulation of AGP in the digestive glands might also be connected with their role in the Ca^2+^ signaling, especially that occurrence of AGP detected with JIM8 antibody increased during the gland activity. However, only future studies will determine whether AGP are indeed linked to the calcium metabolism in the *Dionaea* glands.

### 4.3. Changes in Digestive Glands after Stimulation

Many authors observed that after feeding or prey stimulation, there are changes in the glandular cell structure of the carnivorous plant glands [16]. This process, which is called aggregation, is characterized by shrinkage of the vacuole accompanied by an increased organelle movement [53]. Such changes (e.g., the disintegration of large vacuoles) also occurred in the digestive glands of *Dionaea* and are thought to be connected to provide space for additional cytoplasmic compartments [13,15,21,53]. The cytological changes are consistent with the changes in the presence of AGP (JIM13) that we observed in the glands at the beginning of the secretory phase. However, not only are the vacuoles, cytoplasm and organelles changing [12,13,21] during gland activity but the cell walls are also modified. According to Schnepf [54], the wall ingrowths reach a peak of complexity during the maximum secretory activity. Interestingly, Schwab et al. [21] also described changes in the wall ingrowths during the functioning of the digestive gland. These authors observed maximum changes in the cell wall and wall ingrowths three or four days after feeding, which they interpreted as the time of maximum secretion. They observed that the cell wall ingrowths were diminished in size and number. However, formation of an enlarged interfacial area was observed that probably contained the secreted material. During the next phase (five to seven days after feeding—the absorption phase), the enlarged interfacial area disappeared, but the wall ingrowths were obvious. The cytological changes are consistent with the changes in the presence of AGP (JIM8) that we observed in the glands at the absorption phase. Moreover, according to Boulogne et al. [13], after three days of feeding, an enlarged periplasmic space was formed (which might be the same structure as the “enlarged interfacial area” that was observed by Schwab et al. [21]). We observed the occurrence of AGP in the digestive *Dionaea* gland during all of the phases of their activity (resting gland, glands during secretion and glands during absorption). There was difference in the AGP occurrence (labeled with JIM8 and JIM13) in the cell walls of the gland secretory cells between the unfed and fed traps.

Taken together, our data show that the AGP are stable wall components of digestive glands of Venus flytrap, however, the role of the cell wall composition in the functioning of the carnivorous plant glands requires detailed study. In the future, we want to compare the glands of other carnivorous plants related to *Dionaea* in terms of the wall composition during the enzyme secretion and nutrient absorption processes.

## 5. Conclusions

In carnivorous plants, AGP were recorded earlier in *Drosera* glands by Samaj et al. [55]. However, this is the first report that presents AGP as cell wall components in the traps of carnivorous plant *D. muscipula*. We demonstrated that:

- There was an accumulation of AGP in the cell walls of the gland secretory cells;
- An accumulation of specific AGP at the site where the wall labyrinth occurred;
- The epitope that is recognized by JIM14 was a useful marker of the digestive glands;
- During experiment significant increase of mean value of fluorescence intensity for AGP detected with JIM8 was observed for unfed (0 h) and traps after 3 d and 7 d of feeding;
- For AGP epitope recognized with JIM13 antibody significant changes of mean value of fluorescence intensity were observed only at the beginning of the experiment (after 2 h of feeding);
- Future studies will have to do a comparison with related species.

## Supplementary Materials

The following are available online at https://www.mdpi.com/article/10.3390/cells11030586/s1, Figure S1: Control reactions of the immunolabeling of the cell wall components; Table S1: Quantification of immunofluorescence labeling; Table S2: Statistical analysis of immunofluorescence labelling.
