# Supplementary material for: Arabinogalactan Proteins in the Digestive Glands of Dionaea muscipula J.Ellis Traps
Source: Cells. 2022 Feb 8;11(3):586. doi: 10.3390/cells11030586 (PMC8833951; doi:10.3390/cells11030586)
Supplement: Supplementary file 1 [file cells-11-00586-s001.zip › cells-1552689-supplementary.pdf]

Figure S1

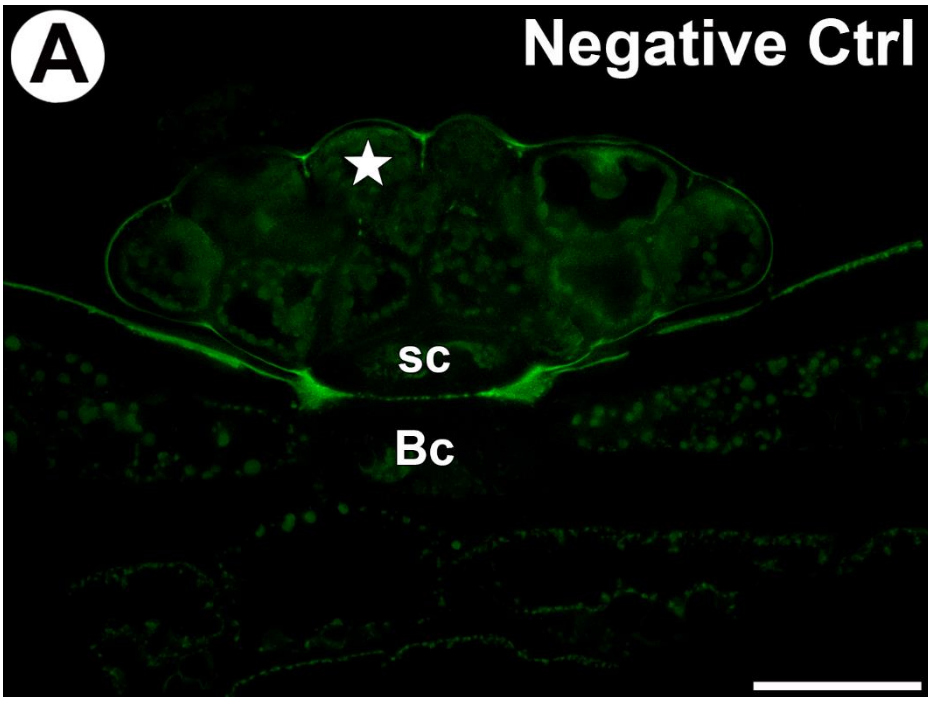

Figure S1. Control reactions of the immunolabeling of the cell wall components. (**a**) The digestive gland; secretory cell (star), stem cell (sc), basal cell (Bc), bar 20 μm.

Table S1

Table S1. Quantification of immunofluorescence labelling. Mean value of AGP fluorescence intensity labelled by JIM14, JIM8, and JIM13 antibodies and in the negative control reaction in secretory cells during 0 and 2 hours, 3, 5, and 7 days after feeding. The regions of interest were selected manually to cover 3 secretory cells from 3 different glands (n=3).

The arabinogalactan epitope recognized by antibody	Time of feeding (hours, days)	Mean value of fluorescence intensity ± SD
**Negative control reaction**		**229.82 ± 13.54**
**JIM14**	0 h	541.66 ± 53.12
	2 h	624.80 ± 30.64
	3 d	598.54 ± 89.9
	5 d	570.09 ± 105.54
	7 d	715.48 ± 102
**JIM8**	0 h	593.91 ± 72.95
	2 h	649.11 ± 106.15
	3 d	609.45 ± 43.54
	5 d	826.92 ± 77.59
	7 d	910.74 ± 151.70
**JIM13**	0 h	509.19 ± 55.49
	2 h	867.12 ± 160.93
	3 d	587.83 ± 49.63
	5 d	607.7 ± 147.32
	7 d	689.85 ± 33.06

Table S2. Statistical analysis of immunofluorescence labelling. Mean value of AGP fluorescence intensity (MFI) labelled by JIM14, JIM8, and JIM13 antibodies in secretory cells in unfed traps (0 h) and in stimulated traps: 2 hours, 3, 5, and 7 days after feeding. The regions of interest were selected manually to cover 3 secretory cells from 3 different glands (n=3). Significance shown in red.

	One -way ANOVA							
	**effect SS**	**effect Df**	**effect MS**	**error SS**	**df**	**error MS**	**F**	**p-level**
**JIM14 MFI**	53213.70	4	13303.43	66774.1	10	6677.411	1.992303	0.171739
**JIM13 MFI**	222549.6	4	55637.41	108476.9	10	10847.69	5.128965	0.016482
**JIM8 MFI**	230260.8	4	57565.20	75468.18	10	7546.818	7.627744	0.004368

**Time of feeding**	**Tukey; JIM8 MFI p < 0.05000**			
	**2 h**	**3 d**	**5 d**	7 d
**0 h**	0.133099	0.999422	0.050568	0.008364
**2 d**		0.183555	0.966077	0.416589
**3 d**			0.070937	0.011571
**5 d**				0.761402

**Time of feeding**	**Tukey; JIM13 MFI p < 0.05000**			
	**2 h**	**3 d**	**5 d**	7 d
**0 h**	0.012260	0.881053	0.773570	0.281973
**2 d**		0.050634	0.072640	0.297274
**3 d**			0.999256	0.751973
**5 d**				0.864306
